# DFT calculations of the structure and stability of copper clusters on MoS_2_

**DOI:** 10.3762/bjnano.11.30

**Published:** 2020-02-26

**Authors:** Cara-Lena Nies, Michael Nolan

**Affiliations:** 1 Tyndall National Institute, University College Cork, Lee Maltings, Dyke Parade, Cork, T12 R5CP, Ireland

**Keywords:** copper (Cu), density functional theory (DFT), 2D materials, molybdenum disulfide (MoS_2_), thin film nucleation

## Abstract

Layered materials, such as MoS_2_, are being intensely studied due to their interesting properties and wide variety of potential applications. These materials are also interesting as supports for low-dimensional metals for catalysis, while recent work has shown increased interest in using 2D materials in the electronics industry as a Cu diffusion barrier in semiconductor device interconnects. The interaction between different metal structures and MoS_2_ monolayers is therefore of significant importance and first-principles simulations can probe aspects of this interaction not easily accessible to experiment. Previous theoretical studies have focused particularly on the adsorption of a range of metallic elements, including first-row transition metals, as well as Ag and Au. However, most studies have examined single-atom adsorption or adsorbed nanoparticles of noble metals. This means there is a knowledge gap in terms of thin film nucleation on 2D materials. To begin addressing this issue, we present in this paper a first-principles density functional theory (DFT) study of the adsorption of small Cu*_n_* (*n* = 1–4) structures on 2D MoS_2_ as a model system. We find on a perfect MoS_2_ monolayer that a single Cu atom prefers an adsorption site above the Mo atom. With increasing nanocluster size the nanocluster binds more strongly when Cu atoms adsorb atop the S atoms. Stability is driven by the number of Cu–Cu interactions and the distance between adsorption sites, with no obvious preference towards 2D or 3D structures. The introduction of a single S vacancy in the monolayer enhances the copper binding energy, although some Cu*_n_* nanoclusters are actually unstable. The effect of the vacancy is localised around the vacancy site. Finally, on both the pristine and the defective MoS_2_ monolayer, the density-of-states analysis shows that the adsorption of Cu introduces new electronic states as a result of partial Cu oxidation, but the metallic character of Cu nanoclusters is preserved.

## Introduction

Since the successful exfoliation of monolayers of graphene by Novoselov et al., 2D materials have gained a large interest in a variety of research areas [1]. These include catalysis [2–3], photonics [4–5], batteries [6], sensors [7–8] and semiconductors and electronics [9–11]. More recently, 2D materials have been explored as copper diffusion barriers in CMOS interconnect structures [12–15]. Furthermore, to enable the use of 2D materials in technology applications, processes have been developed to grow 2D materials via chemical vapour deposition (CVD) [16–17] and atomic layer deposition (ALD) [18–19]. The films prepared via thin film deposition were comparable in performance to materials obtained via exfoliation. However, the scalability of CVD and ALD processes makes 2D materials grown via these methods more realistic for a wider range of applications [4].

Transition metal dichalcogenides (TMDs) are of particular interest as they exhibit a large variety of properties. TMDs such as MoS_2_ are intrinsic semiconductors, unlike graphene, and have thus garnered significant interest in the electronics industry [4]. Often, the properties of the monolayer are different from those of the bulk materials. For example, MoS_2_ has an indirect bandgap in its bulk structure, while it exhibits a direct bandgap as a monolayer [20]. The extensive interest in MoS_2_ can be in part attributed to its favourable properties compared to graphene, as well as the fact that it occurs naturally [21].

There have been numerous computational studies of MoS_2_ and other 2D materials [9,22–23], many of which have examined the adsorption of, or doping with, various elements including transition metals [3,9,24–28], alkali and alkaline-earth metals [29–31] as well as non-metals such as H, B, C, O and N [31]. Work involving atom adsorption on 2D materials can generally be divided into two categories: single-atom adsorption [26,29–31] and adsorption of larger structures such as nanoparticles [25] or metal chains [24].

Studies of single-atom adsorption have focused on screening the stability of a range of elements at TMD monolayers. As an example, Wang et al. [26] studied the adsorption energy, stable geometries, magnetic and electronic properties of first-row transition metal atoms adsorbed on a monolayer of MoS_2_. All metals studied adsorb strongly on the MoS_2_ monolayer, except for Zn, whereby the adsorption energy depends on the identity of the adsorbed element; this is proposed to be related to the number of d electrons. In general, the atoms prefer to adsorb above a Mo atom, however Sc, Ti and Mn prefer a hollow site inside the Mo–S hexagon. Overall, it was concluded that the band structure and magnetic properties of 2D MoS_2_ can be modified by adsorbing different transition metals [26].

Li et al. [29] and Makaremi et al. [31] also examined adsorption of a variety of elements including alkali and alkaline-earth metals as well as non-metals such as H, C and O on MoS_2_ and C_3_N. Both studies aimed to screen different ways in which the monolayers could be functionalised, depending on the type of atom that is adsorbed. Li et al. [29] find that normally semiconducting MoS_2_ monolayers can be tuned to exhibit metallic or semi-metallic behaviour depending on the adatom type. All atoms studied had favourable adsorption energies. Mg had the weakest interaction with a computed adsorption energy of 0.60 eV, while Mn had the strongest interaction, with a computed binding energy of 6.57 eV. The preferred adsorption site depends on the adsorbed atom. The majority of atoms prefers to adsorb above Mo, including Cu, C and Mg. Mn and Ag prefer to adsorb at a hollow site of the Mo–S hexagon, and Au and O adsorb atop the S atoms. Similarly, Makerami et al. [31] find that the functionalisation of semiconducting C_3_N monolayers with non-metallic and semi-metallic elements leads to metallic behaviour. Interestingly, not all metallic adatoms were found to induce metallic behaviour. Metals such as Mg, Cr and Zn are unable to alter the surface from semiconducting to metallic. This selective alteration of the electronic properties through functionalisation makes 2D monolayers attractive candidates for various applications, such as photocatalysis, sensors and electronic devices.

Other work from Ersan et al. [30] focused on adsorption structures of Li at Se-doped MoS_2_, to study the suitability of the system for application in Li-ion batteries. Li adatoms prefer to adsorb above an Mo atom in the monolayer, and cause the system to become metallic once adsorbed. External strain was found to strongly modify the binding energy, with binding decreasing as tensile strain increases. While Li can diffuse through the monolayer, the activation energy required is greater than 1 eV and increases with decreasing Se content. Investigation of on-surface diffusion showed that the magnitude of the activation energies is suitable for the targeted battery applications [30].

Studies of the adsorption of larger structures include the adsorption of 1D metal chains of Cu, Ag and Au [24] on a monolayer of graphene, in which two different conformations of metal chains, namely zig-zag and armchair, are studied. The metal chains physisorb onto the monolayer, and calculations using different van der Waals (vdW) corrections show that the adsorption is driven by vdW interactions. The metal chains prefer to adsorb in the armchair conformation and cause a break of the hexagonal symmetry of graphene. Despite slightly contradictory results depending on the computational setup, the authors conclude that the adsorption of noble metal chains allows for a small opening of the bandgap of graphene, although they are unable to interpret the exact mechanism by which this occurs. The adsorption of 29 atom nanoparticles of Cu, Ag and Au on a MoS_2_ monolayer is presented by Rawal et al. [25] to study the effect of defects in MoS_2_ on the catalytic activity of the supported nanoparticles. They observe that the magnitude of binding energy and charge transfer follows the trend Cu *>* Ag *>* Au. On the pristine surface the binding energies of the nanoparticles are 5.4 eV for Cu, 4.2 eV for Ag and 4.5 eV for Au. The presence of a complete row of sulfur vacancies enhances the adsorption energy of the nanoparticles for all three metals, increasing it to 7.1 eV, 7.0 eV and 6.0 eV for Cu, Ag and Au, respectively. It also increases the charge transfer from the nanoparticle to the MoS_2_ monolayer by approximately 1 electron for Cu and Ag and by 0.6 electrons for Au. Studying the adsorption and dissociation of O_2_ on the nanoparticle demonstrated that the MoS_2_ support improves the catalytic activity of the nanoparticles, compared to an unsupported nanoparticle, in particular when the monolayer is defect-rich.

MoS_2_ is known to be naturally high in defects [21,32], in particular S vacancies. It has been predicted that S vacancies in a MoS_2_ monolayer are most stable when they occur in a row, with a decrease in the vacancy formation energy as the number of vacancies increases [2]. Experimental methods for controlling the formation of sulfur vacancies in the MoS_2_ monolayer have also been developed [33], and this would allow for the targeted use of S vacancies to enhance desired properties such as adsorption energy.

In this study we aim to fill the gap in the literature between the adsorption of single Cu atoms and the adsorption of larger structures from the publications discussed above. We choose the Cu–MoS_2_ ML system due to its potential significance for the electronics industry as a copper diffusion barrier [12–15]. Studying small Cu*_n_* (*n* = 1–4) structures allows us to investigate the first stages in the nucleation of a Cu film on MoS_2_ monolayers, as well as the fundamental copper–TMD interactions, and thus gain significant insights into the range of stable configurations for Cu adsorption on MoS_2_. In addition, we investigate the effect of a single S vacancy on the adsorption energy and geometry of single Cu adatoms and the Cu_4_ clusters. The results of this investigation show that the stability of small Cu*_n_* clusters on a MoS_2_ ML is driven mainly by Cu–Cu interactions and not dependent on whether the cluster is 2D or 3D. Further, the density-of-states (DOS) analysis shows the emergence of mid-gap states, indicating that the system is changing from semiconducting to metallic as Cu atoms are adsorbed, making it suitable for application as a Cu diffusion barrier.

## Computational Methods

**Figure 1:**
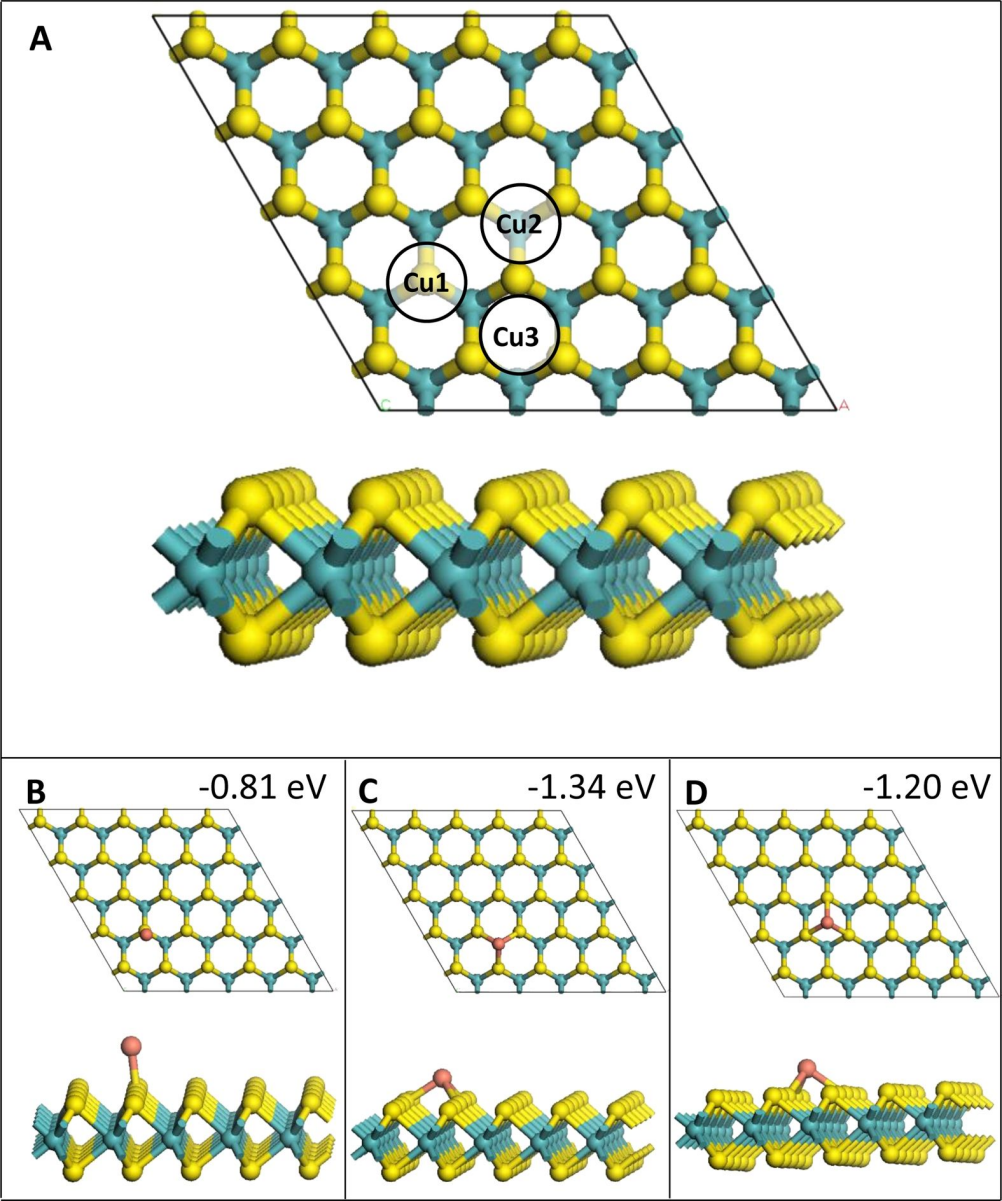
(A) Adsorption sites on a MoS_2_ monolayer. (B–D) Relaxed structures after adsorption of one Cu atom on the monolayer at sites **1**, **2** and **3**, respectively. Yellow = S, teal = Mo, orange = Cu.

All calculations, for bulk MoS_2_ and the 2D monolayer, were carried out with density functional theory (DFT) using the Vienna ab initio simulation package (VASP) version 5.4 [34]. It uses 3D periodic boundary conditions and the spin-polarized generalized gradient approximation (GGA) using the Perdew–Burke–Ernzerhof (PBE) approximation to the exchange–correlation functional [35]. The valence electrons are described explicitly using a plane wave basis set with an energy cut-off of 450 eV. The valence electron configurations used for this study are Mo = 5s^1^4d^5^, S = 3s^2^3p^4^ and Cu = 4s^1^3d^10^. The core–valence electron interactions are described using the projector augmented wave potential (PAW) [36]. In the geometry relaxation calculations, all forces acting on the atoms were converged to within 0.02 eV/Å. The bulk structure of MoS_2_ used in this study was chosen from the “Materials Project” database [37]. The bulk material contains two layers of MoS_2_. The geometry was then optimised by relaxing cell volume, cell shape and ionic position simultaneously, using an energy cut-off of 600 eV, as well as a Monkhorst–Pack K-point sampling grid of (6 × 6 × 12). The computed equilibrium lattice parameters for this setup are *a* = 3.16 Å, *b* = 3.05 Å, *c* = 12.29 Å and α = β = 90.00°, γ = 63.65°. To create a model for the MoS_2_ monolayer (ML), the bottom layer was removed and the supercell was expanded five times in the MoS_2_ plane to create the (5 × 5) supercell shown below in Figure 1A. The ML supercell and all models of Cu adsorption were generated using the atomic simulation environment (ASE) package [38]. The atomic charges were computed from the Bader charge partitioning scheme [39–40].

To understand the binding of Cu to the MoS_2_ monolayer, three different energies were computed.

1. Binding energy per Cu atom:

[1] $$E_{\text{bind/atom}}=\frac{\left(E_{\text{total}}-E_{\text{monolayer}}-nE_{\text{Cu}\_\text{atom}}\right)}{n}$$

*E*_total_ is the total energy of the relaxed Cu*_n_* (*n* = 1–4) adsorbed on MoS_2_. The energy of a single gas phase Cu atom (*E*_Cu_atom_) is multiplied by *n*, the number of Cu atoms in adsorbed Cu*_n_*.

2. Binding energy with reference to a free Cu*_n_* cluster:

[2] $$E_{\text{bind}}=E_{\text{total}}-E_{\text{monolayer}}-E_{\text{Cu}\_\text{cluster}}$$

where *E*_Cu_cluster_ is the energy of the most favourable Cu*_n_* nanocluster structure in vacuum. For two atoms, this is a Cu_2_ dimer, for three atoms it is a triangle and for four atoms it is a tetrahedral configuration.

3. Addition energy:

[3] $$E_{\text{add}}=E_{\text{total}}-E_{\text{monolayer+}(n-1)\text{Cu}}-E_{\text{Cu}\_\text{atom}}$$

where *n* is the number of Cu atoms. This models adding a Cu atom to an existing adsorbed cluster with (*n* − 1) Cu atoms.

For the calculations involving MoS_2_ with an S vacancy, the vacancy formation energy was calculated based the reaction H_2_ + MoS_2_→ MoS_2−_*_x_* + H_2_S, where *x* indicates that S vacancies are present. The vacancy formation energy is then calculated as:

[4] $$E_{\text{form}}=\left(E_{{\text{MoS}}_{2-x}}+E_{{\text{H}}_{\text{2}}\text{S}}\right)-\left(E_{{\text{MoS}}_{\text{2}}}+E_{{\text{H}}_{\text{2}}}\right)$$

In this case, the computed vacancy formation energy is −6.16 eV.

## Results and Discussion

### Cu adsorption

Three different adsorption sites for a single atom, denoted as **1**, **2** and **3**, are present on the MoS_2_ monolayer, as shown in Figure 1A. Site **1** has a Cu atom adsorbed directly atop a S atom. Site **2** has Cu binding to three S atoms directly above an Mo atom and site **3** has Cu binding to three S atoms, but with no Mo atom underneath.

To investigate how Cu begins to nucleate on a monolayer of MoS_2_, we start by adsorbing small Cu*_n_* (*n* = 1–4) species on a MoS_2_ monolayer. All binding energies for the different structures calculated from Equation 1 are shown in Table 1. Binding energies calculated with Equation 2 are shown in Table 2 and addition energies are shown in Table 3.

**Table 1:** Computed binding energies for Cu_1_, Cu_2_, Cu_3_ and Cu_4_ on a MoS_2_ ML for different atom configurations using Equation 1. For the “non-equivalent” configurations of Cu_2_, the column “site **1**” has atoms at sites **1** and **2**, “site **2**” has atoms at **1** and **3** and “site **3**” has atoms at **2** and **3**.

number of Cu atoms	adsorption configuration	*E*_bind_/atom [eV]		

		site **1**	site **2**	site **3**

1	—	−0.81	−**1.32**	−1.18
				
2	neighbouring	−0.84	−1.54	−**1.58**
	separated	−0.79	−1.34	−1.25
	non-equivalent sites	−1.09	−1.00	−1.33
				
3	line	−1.34	−1.47	—
	off-set	−0.82	−1.47	—
	triangle	−0.85	−1.56	−1.64
	3D triangle	−**1.85**	−**1.84**	−1.80
				
4	line	−1.41	−1.48	−1.35
	rhombus	−**2.01**	−1.87	−1.31
	3D rectangle	−1.87	−1.87	−1.77
	tetrahedral	−1.98	−1.86	−1.83

**Table 2:** Computed binding energies for Cu_1_, Cu_2_, Cu_3_ and Cu_4_ on MoS_2_ for different atom configurations from Equation 2. For the “non-equivalent” configurations of Cu_2_, the column “site **1**” has atoms at sites **1** and **2**, “site **2**” has atoms at **1** and **3** and “site **3**” has atoms at **2** and **3**.

number of Cu atoms	adsorption configuration	*E*_bind_ [eV]		

		site **1**	site **2**	site **3**

1	—	−0.81	−**1.32**	−1.18
				
2	neighbouring	−0.88	−2.27	−**2.36**
	separated	−0.77	−1.88	−1.69
	non-equivalent sites	−1.38	−1.20	−1.85
				
3	line	−1.08	−1.46	—
	off-set	0.49	−1.45	—
	triangle	0.39	−1.74	−1.96
	3D triangle	−**2.59**	−**2.59**	−2.46
				
4	line	−1.63	−1.92	−1.41
	rhombus	−**4.04**	−3.47	−1.24
	3D rectangle	−2.43	−3.48	−3.06
	tetrahedral	−3.92	−3.43	−3.31

**Table 3:** Computed addition energies for each configuration (Cu*_n_*_−1_ + Cu_1_ → Cu*_n_*) calculated using Equation 3.

number of Cu atoms	configuration	*E*_add_ [eV]		

		site **1**	site **2**	site **3**

2	neighbouring	−0.87	−1.75	−**1.98**
	separated	−0.77	−1.36	−1.31
	non-equivalent sites	−1.37/−0.86	−1.75/−0.82	−1.34/−1.48
				
3	line	−2.35	−1.34	—
	off-set	−0.78	−1.32	—
	triangle	−0.88	−1.62	−1.75
	3D triangle	−**3.86**	−2.46	−2.25
				
4	line	−1.60	−1.52	—
	rhombus	−**5.48**	−2.79	−0.33
	3D rectangle	−0.89	−1.95	−1.65
	tetrahedral	−5.37	−2.75	−2.41

**Figure 2:**
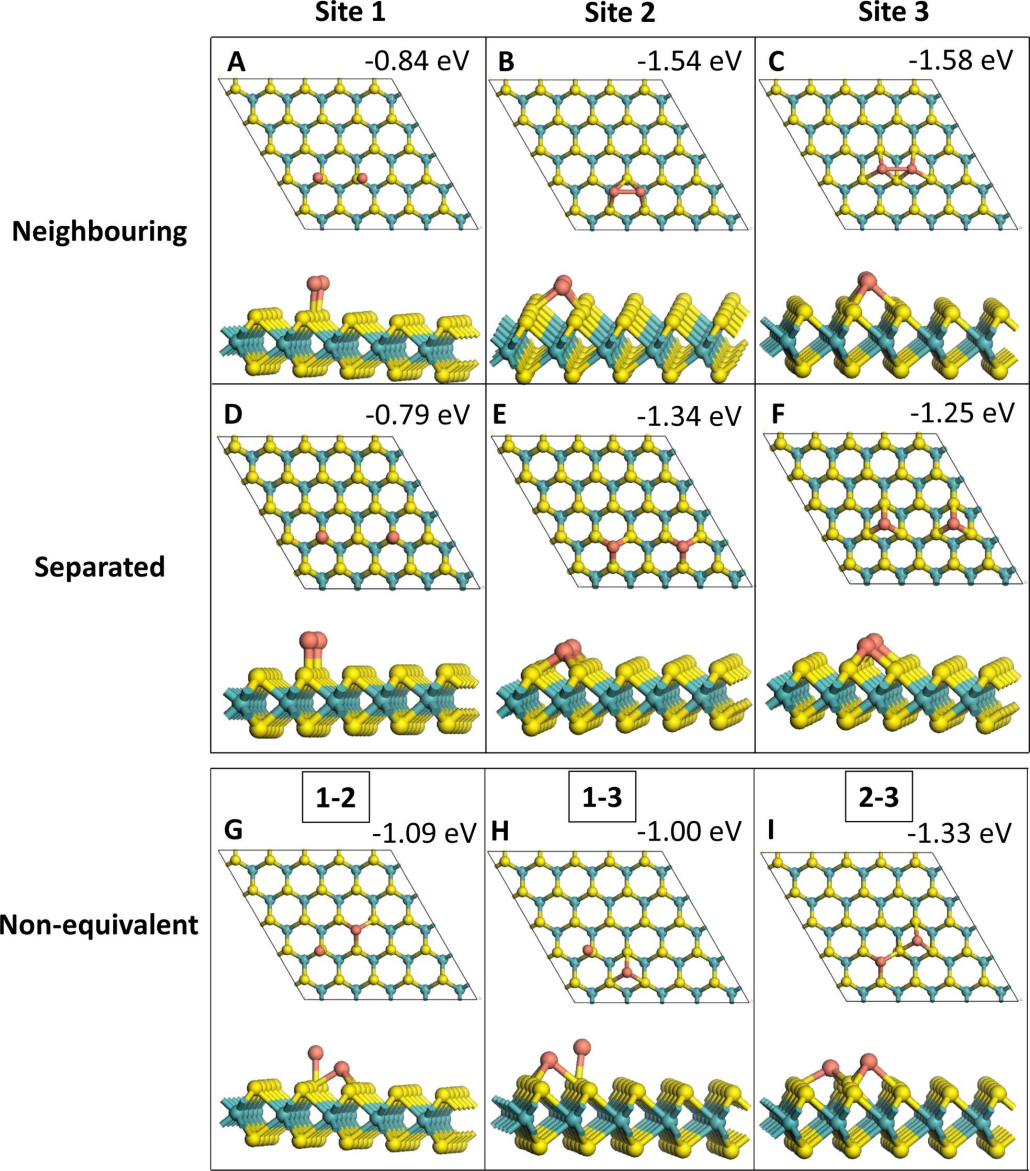
Adsorption configurations with two Cu adatoms. (A) through (F) show adatoms at equivalent sites, while (G), (H) and (I) show combinations of two atoms adsorbed at different sites on the MoS_2_ ML.

**Figure 3:**
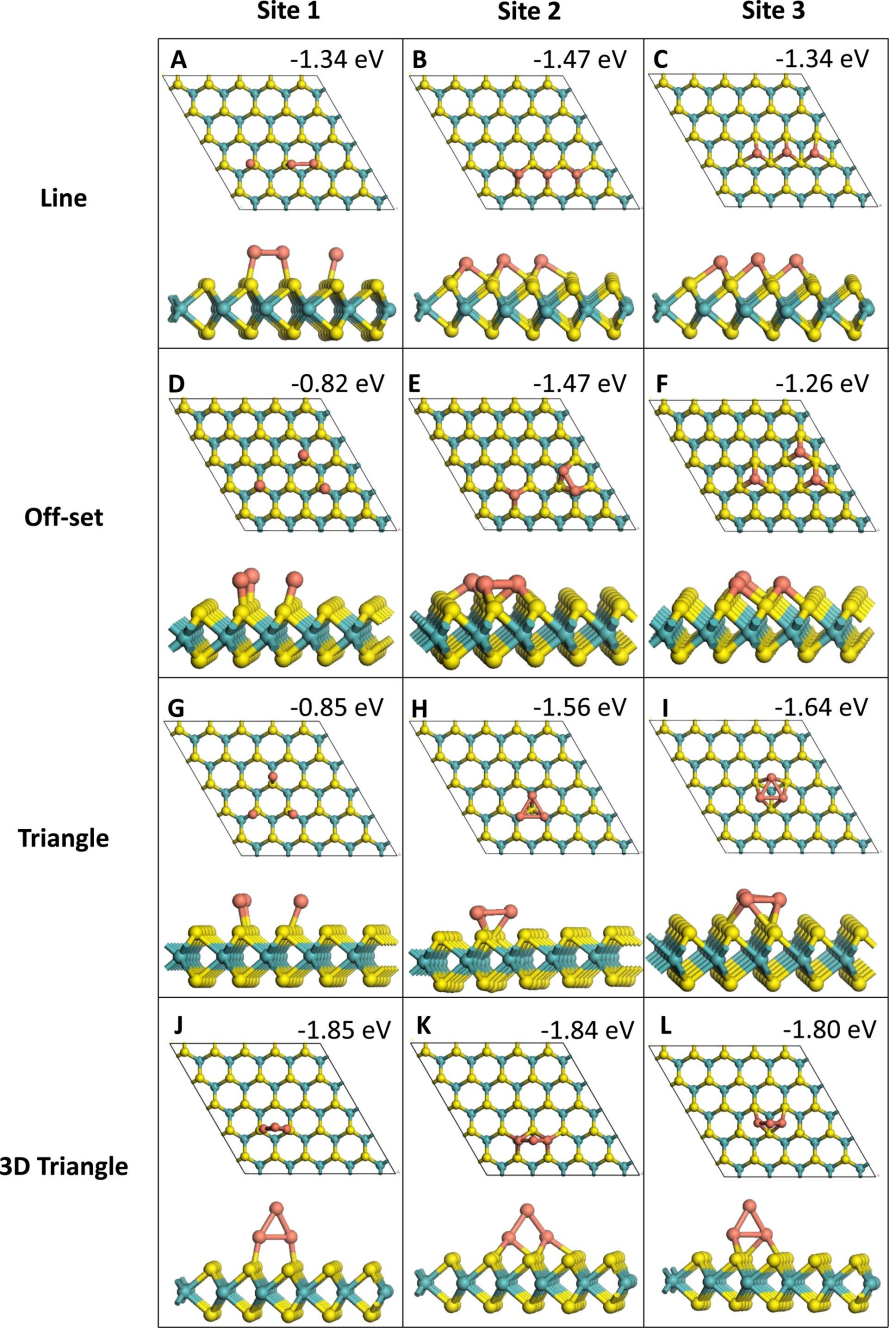
Relaxed structures after adsorption of Cu_3_ structures.

**Figure 4:**
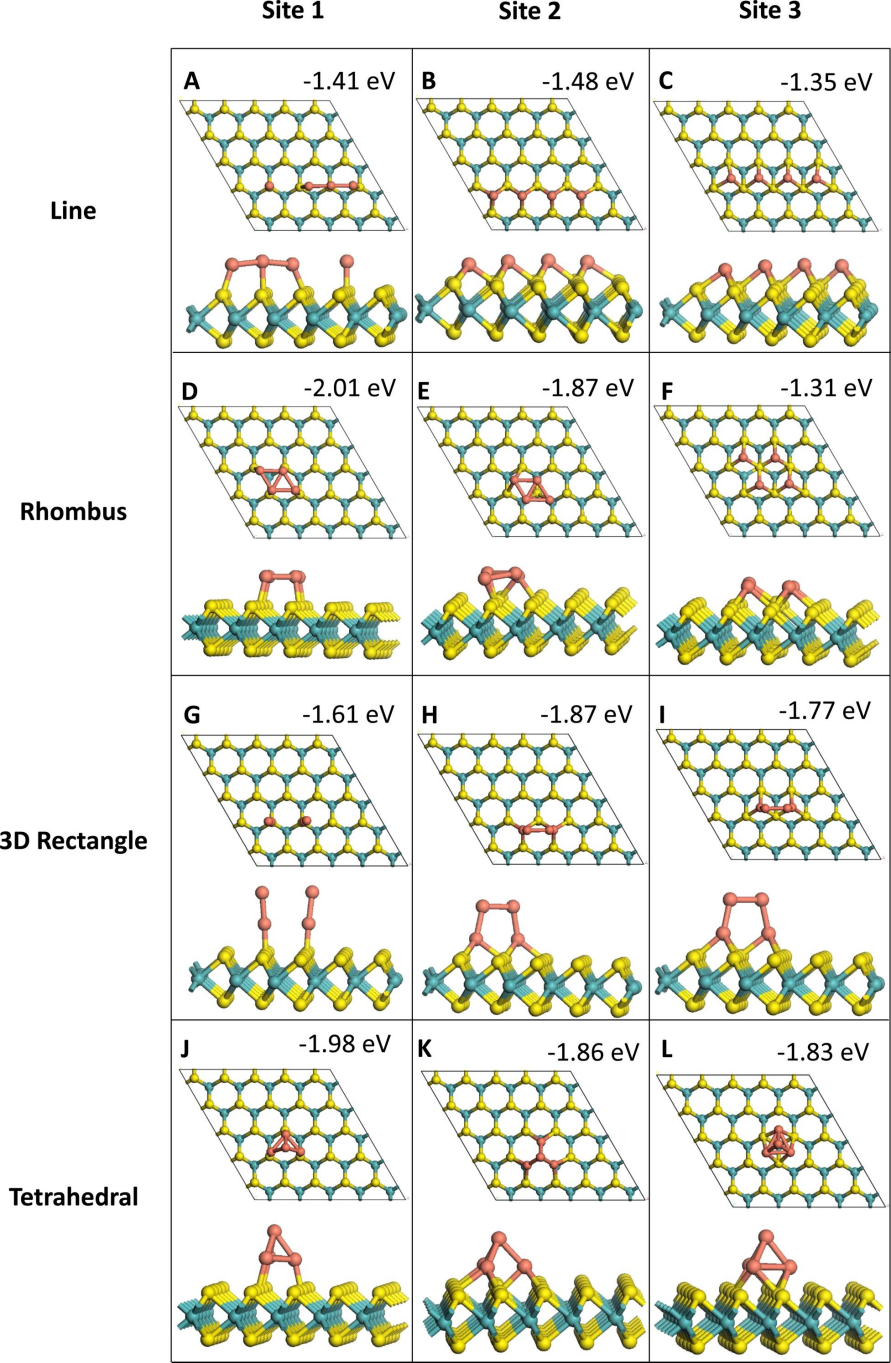
Relaxed structures after adsorption of Cu_4_ structures.

In the 2D adsorption structures all Cu atoms are bound to the MoS_2_ ML while in the 3D adsorption structures at least one of the Cu atoms is not bound to MoS_2_. All relaxed Cu*_n_* (*n* = 1–4) geometries with one, two, three and four Cu atoms are shown in Figure 1, Figure 2, Figure 3 and Figure 4, respectively. The binding energies shown in these images are the binding energies per atom from Equation 1.

When a single Cu atom adsorbs at each of the three adsorption sites, Cu binds exothermically with adsorption energies of −0.81, −1.32 and −1.18 eV at sites **1**, **2** and **3**, respectively. Site **2** is the most favourable site for a single Cu atom. This is most likely due to the particular geometry, as it is a continuation of the MoS_2_ structure, with Cu on a Mo site (see Figure 1C). This order of stability for single-atom adsorption matches results previously published by Li et al. [29] and Wang et al. [26] for Cu adsorption on MoS_2_.

Two Cu adatoms were adsorbed in three different configurations, i.e., as a Cu_2_ species with each Cu atom on an equivalent site, as two separated Cu adatoms and at neighbouring but non-equivalent sites. At site **1**, whether the Cu atoms adsorb as a Cu_2_ or as two separated adatoms makes little difference for the stability; the difference in the binding energy is only 0.05 eV. The addition energy of the second Cu atom is similar to the binding energy in both cases, indicating that adding a second atom yields approximately the same energy gain as the adsorption of the first atom. For the adsorption of two Cu atoms at sites **2** and **3** on the MoS_2_ ML, it is more favourable by up to 0.3 eV to adsorb as Cu_2_ compared to separated adatom adsorption. This is also reflected in the addition energy for Cu to Cu_2_, as this is slightly more negative than both the binding energy per atom for this configuration as well as the binding energy of a single atom at each site.

Out of the non-equivalent Cu adatom adsorption modes, Cu_2_ as a combination of a Cu adatom at site **2** and one at site **3** was most favourable, because sites **2** and **3** are both more favourable than a Cu adatom at site **1**. The addition energies were computed for addition of an atom at both sites, with a Cu atom already at the other site. With an atom adsorbed at site **1**, adding a second atom at sites 2 or 3 gives an energy gain of −1.37 and −1.75 eV, respectively, while adding an atom at site **1** gives an addition energy of −0.86 eV if the first atom is at site **2** and −0.82 eV if the adatom is at site **3**. Thus, the more favourable addition energies are observed when adding an atom to the more favourable adsorption sites. Overall, adsorbing Cu at equivalent sites instead of non-equivalent sites yields a larger increase in binding energy per atom, except for the less stable site **1**, where the combination with a more favourable adsorption site causes an increase in binding energy per atom.

Four different configurations were relaxed for the adsorption of three adatoms. Of these, three configurations are 2D and one is a 3D configuration in which two Cu atoms bind to MoS_2_. The computed binding energies show that the 3D configuration is the most stable of the four (see Table 1). Depending on the exact adsorption site and adsorption configuration, the 3D Cu_3_ adsorption structure is more stable by 0.16 to 1.03 eV, compared to the various 2D structures. While site **1** continued to be less favourable than sites **2** and **3** for the 2D configurations, for the 3D adsorption mode this difference disappears, with similar binding energies for all sites, suggesting that once 3D clusters begin to form on MoS_2_ the binding energy is not influenced by the adsorption site. It is of note that the atoms remain at the site where they were originally adsorbed throughout relaxation. This is also apparent from the computed addition energies. The addition of an atom to Cu_2_ to form a 3D Cu_3_ cluster is very favourable, with computed addition energies lying between −2.25 and −3.86 eV. The addition energy of −3.86 eV for site **1** reflects that this configuration is much more stable than any other three-atom configuration at site **1**.

Finally, for the adsorption of four Cu atoms, four configurations were examined. Two configurations are flat (2D) and two configurations are 3D nanoclusters. For the flat adsorption structures, the four Cu atoms are adsorbed in a linear configuration, with Cu–Cu distances of around 3.2 Å, depending on the adsorption site, or in a 4-membered flat structure. In the 3D clusters, one configuration is a rhombus with two Cu atoms bound to MoS_2_ and the other 3D configuration is a tetrahedron with three atoms bound to MoS_2_ and an apex atom bound to the triangular base. Interestingly, for the 2D structures, binding of Cu atoms at site **1** becomes more favourable than binding of Cu atoms at site **3**. As for the Cu_3_ structures, the atoms remain at their original adsorption site throughout the relaxation for all Cu_4_ structures. For the linear Cu_4_ configuration the binding energy at site **1** is only 0.06 eV per Cu more negative than at site **3** and 0.07 eV less negative than at site **2**, indicating little difference in stability. We see that this is the weakest adsorption configuration of Cu_4_ at site **1**. This is presumably a result of fewer Cu–Cu bonds, as there is little variation in the Cu–Cu bond lengths, which range from 2.24 to 2.41 Å for structures at site **1**. However for the 2D rhombus adsorption configuration, site **1** is the most favourable site, with a binding energy that is 0.14 eV more negative than that at site **2** and 0.70 eV more negative than the binding energy at site **3**. This is likely due to the Cu–Cu interactions, which are in fact absent when adsorbing the rhombus structure at site **3**, the weakest overall Cu_4_ adsorption configuration, due to long Cu–Cu distances of 3.0 to 3.5 Å.

The rhombus configuration of Cu_4_ at site **1** has a computed addition energy of −5.48 eV relative to Cu_3_, which is the most favourable addition energy calculated, while the addition energy for the tetrahedral configuration at site **1** is similar with a value of −5.37 eV. This indicates that Cu prefers this Cu_4_ structure with Cu–Cu interactions compared to the corresponding Cu_3_ structure at site **1**, which has no Cu–Cu interactions. Site **2** has favourable addition energies for all four configurations. By contrast, site **3** has a much less favourable addition energy of only −0.33 eV for the rhombus configuration. For both 3D configurations, all three sites have very similar adsorption energies, further indicating the lack of influence of the adsorption site on the stability, with an energy difference between the 3D structures of around 0.1 eV. The addition energies for the tetrahedral configuration are much larger than those for the upright 3D adsorption configuration, with a difference of about 0.8 eV for sites **2** and **3** and a difference of 4.48 eV for site **1**. The 3D structures are more favourable than the 2D structures at site **3**. However for sites **1** and **2**, the rhombus configuration has a binding energy that is approximately the same as that of the tetrahedron. The instability of the rhombus at site **3** compared to the same configuration at sites **1** and **2** is likely due to the lack of Cu–Cu bonds, which are present in structures at sites **1** and **2** but not at site **3** (see Figure 4F).

In general, the binding energy relative to free Cu*_n_* clusters (Equation 2) shows the same trends as the binding energies per Cu atom from Equation 1. The off-set and triangle configurations for Cu_3_ at site **1** (as shown in Figure 3D,G) resulted in a positive binding energy when calculated with Equation 2, indicating that they are unstable. However, this is most likely due to the most favourable gas phase three-atom cluster having a different structure from that taken upon adsorption on the MoS_2_ ML. When calculating the stability using adsorbed Cu_3_ clusters, the structures were found to have relatively low binding energies of −0.82 and −0.85 eV.

It is of note, that even though for single-atom adsorption, site **1** is the least favourable adsorption site, the most favourable binding energies are found for the rhombus and tetrahedral Cu_4_ configuration at site **1**. These also had the most favourable addition energies, reflecting the large increase in stability of configurations at site **1** as subsequent Cu atoms are added. Overall, the binding energy per copper atom increases as more atoms are added, which is most likely due to the formation of Cu–Cu bonds and more Cu–surface bonds. The addition of Cu atoms also becomes more favourable, with the exception of the rhombus configuration of Cu_4_ at site **3**.

This analysis indicates that the relative stability of an adsorption configuration of a Cu*_n_* species appears to be generally determined by the presence or absence of Cu–Cu bonding. Those configurations with a larger number of Cu–Cu bonds tend to have a more favourable binding energy. This becomes particularly clear for Cu_4_, where all structures other than the 3D tetrahedron are less favourable at site **3** than at sites **1** and **2**. The distance from site **3** to another equivalent site is longest at approximately 3.2 Å, which then results in very long Cu–Cu bonds, or in the case of Cu_4_ line and rhombus structures, no Cu–Cu bonds.

Cu–S distances also vary with adsorption site and are found to be shortest for Cu atom adsorption at site **1**, with Cu–S distances in the range of 2.16 to 2.31 Å. This is due to the Cu atom being adsorbed directly atop the S atom. In comparison, Cu–S bond lengths are somewhat longer at the other sites, ranging from 2.23 to 2.54 Å at site **2** and 2.26 to 2.58 Å at site **3**. Overall, the bond lengths appear to be determined by the adsorption site and do not have a strong effect on the strength of adsorption.

Bader charge analyses of Cu atoms and adjacent Mo and S atoms are shown in Table 4, Table 5 and Table 6. It shows that Cu atoms that bind directly with multiple sulfur atoms on the MoS_2_ layer, for example at sites **2** and **3**, are clearly oxidised, with computed Bader charges of 10.6 electrons (the number of valance electrons in Cu is 11). In contrast, those Cu atoms that bind to a single sulfur atom, e.g., site **1**, are less oxidised with a computed Bader charge of 10.8 electrons. Cu atoms that do not bind to the surface are metallic, with computed Bader charges of 11.0 to 11.1 electrons. There are no significant changes in the computed Bader charges of Mo and S atoms in the monolayer, which are ca. 4.9 and 6.5 electrons, respectively. Analysis of the charge density difference after Cu*_n_* adsorption, confirms the observations made from the Bader analysis. Charge density is localised mainly at the adsorbed Cu atoms, with less charge density at those Cu atoms that are not bound to the surface. Some charge density is also observed in the surface S atoms interacting with Cu atoms, as well as those Mo atoms that are bound to the interacting S atoms. Figure S5A–G of Supporting Information File 1 shows the charge density difference of the most favourable Cu*_n_* adsorption configurations.

**Table 4:** Computed Bader charge (*Q*) on each Cu atom for Cu_1 and Cu_2.

site	adsorption configuration	*Q*(Cu_1) [electrons]	*Q*(Cu_2) [electrons]

1	single atom	10.81	—
2	**single atom**	10.62	—
3	single atom	10.62	—
			
1	neighbouring	10.81	10.81
2	neighbouring	10.73	10.74
3	**neighbouring**	10.83	10.84
			
1	separated	10.85	10.86
2	separated	10.62	10.61
3	separated	10.62	10.60
			
1-2	non-equivalent	10.81	10.64
1-3	non-equivalent	10.82	10.62
2-3	non-equivalent	10.49	10.68

**Table 5:** Computed Bader charge (*Q*) at each Cu atom for different adsorption configurations of three Cu atoms. In 3D structures, Cu_1 and Cu_2 are interacting with the monolayer, while Cu_3 is at the apex of the triangle.

site	configuration	*Q*(Cu_1)	*Q*(Cu_2)	*Q*(Cu_3)

1	line	10.82	10.87	10.85
2	line	10.65	10.77	10.64
3	line			
				
1	off-set	10.84	10.81	10.82
2	off-set	10.63	10.73	10.74
3	off-set			
				
1	triangle	10.81	10.80	10.81
2	triangle	10.76	10.75	10.74
3	triangle	10.77	10.75	10.76
				
1	**3D triangle**	10.76	10.76	11.00
2	**3D triangle**	10.67	10.67	11.07
3	3D triangle	10.72	10.73	11.02

**Table 6:** Computed Bader charge (*Q*) at each Cu atom for different adsorption configurations with four Cu atoms. For the 3D rectangle configurations, Cu_1 and Cu_2 are interacting with the monolayer and Cu_3 and Cu_4 are adsorbed atop of Cu_1 and Cu_2. For the tetrahedron, Cu_4 is at the apex, while Cu_1, Cu_2 and Cu_3 are all interacting with the MoS_2_ monolayer.

site	configuration	*Q*(Cu_1)	*Q*(Cu_2)	*Q*(Cu_3)	*Q*(Cu_4)

1	line	10.78	10.84	10.97	10.84
2	line	10.62	10.77	10.77	10.61
3	line	10.61	10.78	10.79	10.62
					
1	**rhombus**	10.90	10.83	10.92	10.80
2	rhombus	10.84	10.85	10.80	10.83
3	rhombus	10.61	10.78	10.79	10.62
					
1	3D rectangle	10.80	10.80	11.11	11.11
2	3D rectangle	10.70	10.71	11.05	11.05
3	3D rectangle	10.71	10.71	11.03	11.04
					
1	tetrahedron	10.87	10.75	10.87	11.00
2	tetrahedron	10.68	10.67	10.69	11.07
3	tetrahedron	10.72	10.71	10.71	11.00

Similarly, the bond lengths between Mo and S atoms in the monolayer do not vary significantly from those in the bare MoS_2_ monolayer after adsorption of Cu atoms. Cu–S bonds can vary from the value in CuS bulk material (2.31 Å [41]) by up to +0.27 Å, in the case of adsorption at site **3**, in particular the Cu_4_ line configuration, or by −0.15 Å, in the case of most of the adsorption configurations at site **1**. Mo–S distances are found to be within +0.09 Å and −0.05 Å of the Mo–S distance in the bare ML (2.41 Å). Thus, no significant structural distortion occurs in MoS_2_ after Cu adsorption. We conclude, that there is no clear correlation between the Cu–S or Mo–S distances and the favourability of a binding site.

**Figure 5:**
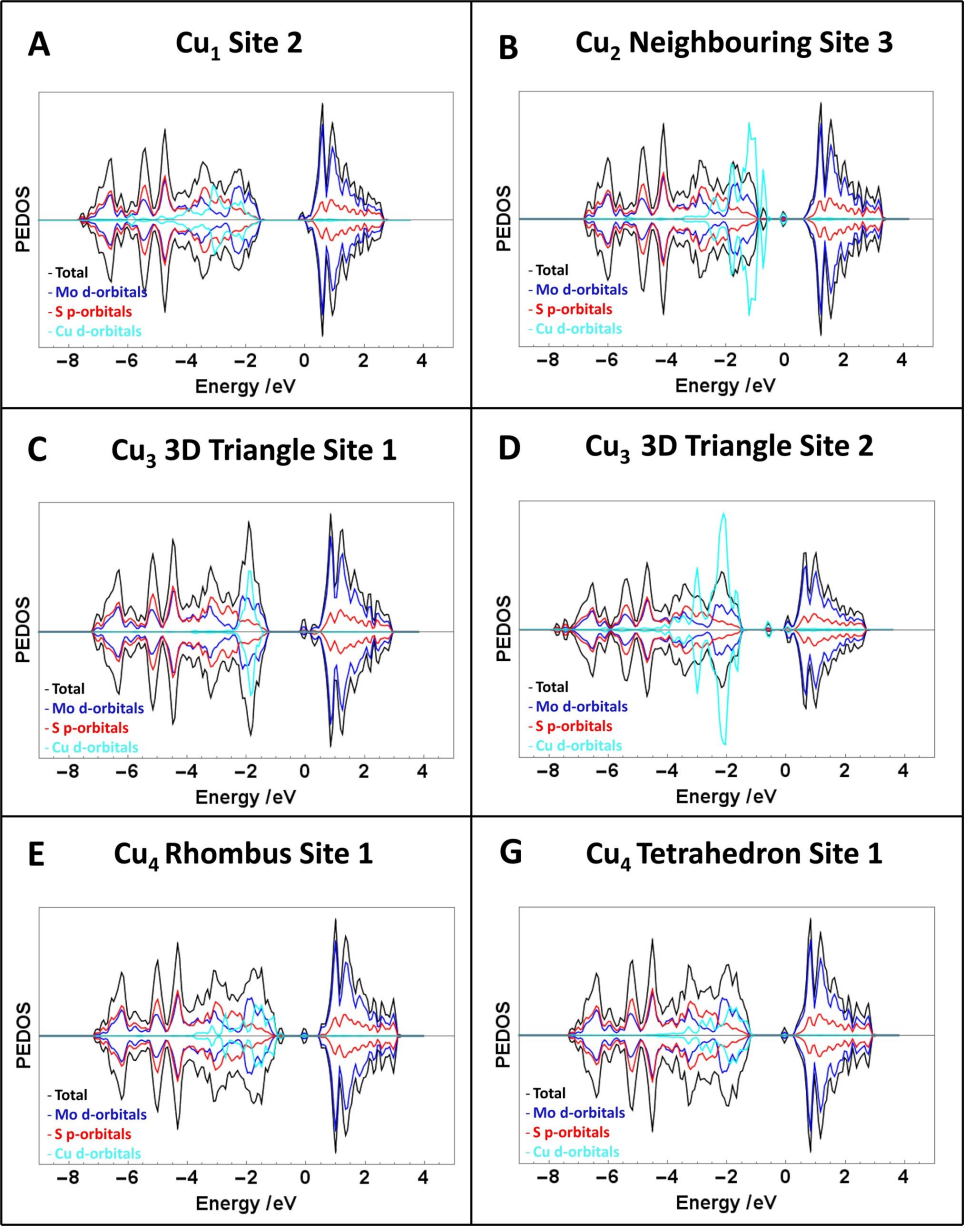
DOS plots of most favourable adsorption configuration for each Cu*_n_* structure on MoS_2_. The contribution of the Cu d orbitals has been increased by a factor of five for the ease of comparison and zero on the “energy” scale is the Fermi level.

Analysis of the density of states (DOS) (see Figure 5 for the most favourable adsorption structures for each Cu*_n_* structure) shows the emergence of mid-gap states as Cu atoms are added to the monolayer. These states arise from the partial oxidation of Cu atoms to produce Cu^+^ cations and include contributions from all three elements. Exceptions to this are the 3D triangle configurations at adsorption sites **1** and **3**, where there is no mid-gap state with all of the Cu contributions at the Fermi level. Density of states plots for all Cu*_n_* structures can be found in Supporting Information File 1, Figures S1–S3.

### Cu adsorption on MoS_2_ with one S vacancy

S vacancies can form in MoS_2_ monolayers with relative ease [2]. Le et al. showed that these vacancies become more stable when a row of vacancies is present in the monolayer. For the purpose of this study we limit ourselves to a single vacancy in the monolayer, giving us a first insight of how Cu adsorption is affected by the presence of a sulfur vacancy. Single Cu atoms and Cu_4_ nanoclusters were adsorbed on the defective MoS_2_ surface with a single sulfur vacancy. While all single-atom adsorption configurations that we investigated are stable, only five of the twelve Cu_4_ nanocluster structures were stable upon relaxation. For those adsorption structures that were not stable we generally find Cu atoms repelled from the surface and an endothermic adsorption energy. An example is shown in Supporting Information File 1, Figure S6 where a Cu atom is repelled from the defective monolayer during the geometry relaxation of a Cu_4_ rhombus structure adsorbed at site **1** and near the sulfur vacancy.

**Figure 6:**
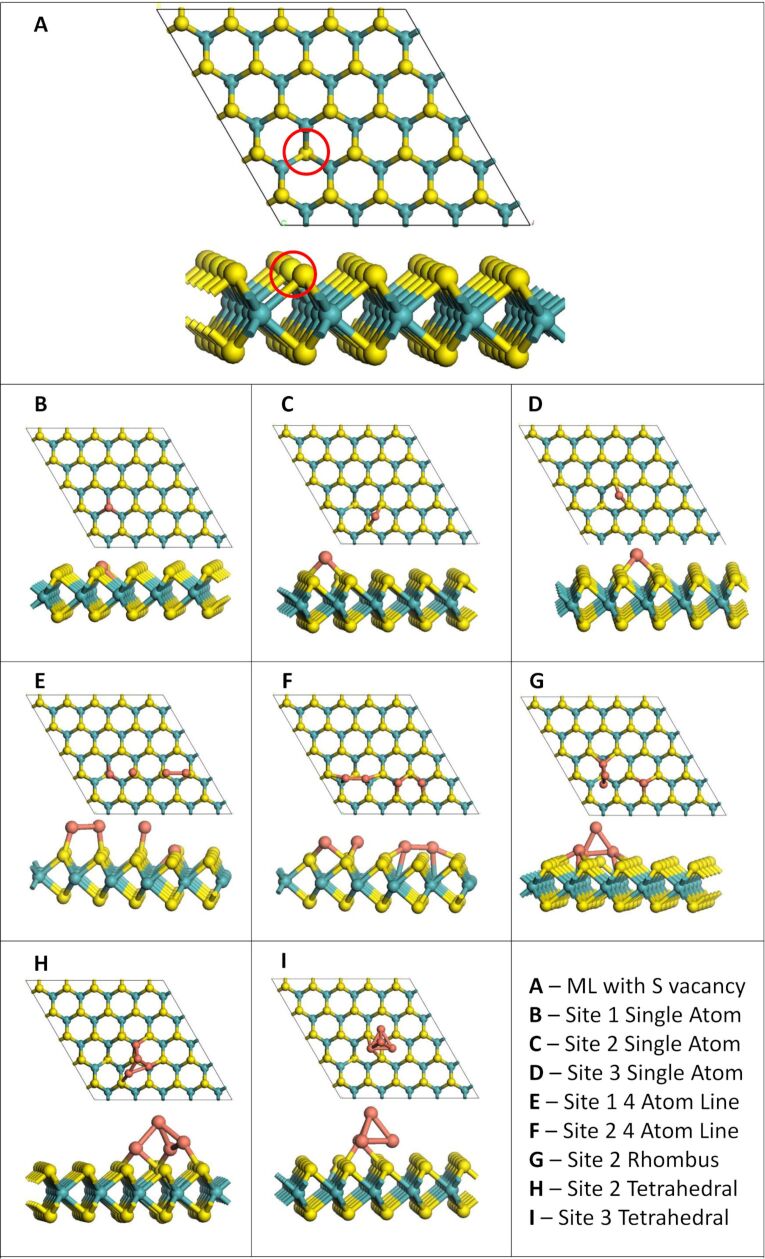
Bare MoS_2_ ML with S vacancy (highlighted in red) and adsorption structures for Cu_1_ and Cu_4_ adsorbed on MoS_2_ with one sulfur vacancy.

The binding energies of Cu and Cu_4_, using Equations 1 and 2, are presented in Table 7 and the stable adsorption geometries are shown in Figure 6. Comparing with the stoichiometric MoS_2_ monolayer, Figure 6, all binding energies are more favourable for adsorption on the defective monolayer. The largest increase in the computed binding energy is 1.95 eV, and this was observed for the single Cu atom adsorption initially at site **1**. Upon relaxation, the adsorbed Cu atom migrates into the vacancy site and replaces the missing S atom. This results in Cu–Mo distances of 2.60 to 2.63 Å and there are no bonds to the surrounding S atoms. In contrast, adsorption of the Cu atom at sites **2** and **3**, away from the vacancy site, results in an increase in the binding energy of only 0.03 and 0.06 eV. Cu–S bonds are ca. 2.18 Å for adsorption at sites **2** and **3**, which is only slightly longer than the Cu–S distance of 2.15 Å for adsorption on the pristine surface. This indicates that the influence of a single sulfur vacancy in the monolayer is localised.

**Table 7:** Computed binding energies per Cu atom of one and four Cu atoms and binding energies of the Cu_4_ nanocluster relative to a free Cu_4_ nanocluster on MoS_2_ with a sulfur vacancy for the adsorption of a single Cu atom and the Cu_4_ nanocluster.

number of Cu atoms	configuration	*E*_bind_/atom [eV]			*E*_bind_ [eV]		
	
		site **1**	site **2**	site **3**	site **1**	site **2**	site **3**

1	—	−2.76	−1.35	−1.24	−2.76	−1.35	−1.24
							
4	line	−1.75	−1.83	—	−3.01	−3.30	—
	rhombus	—	−2.26	—	—	−5.02	—
	tetrahedral	—	−2.05	−1.93	—	−4.20	−3.31

For the adsorption of the four-atom nanocluster, the largest increase in the computed binding energy is found for the initial flat rhombus configuration at site **2**. Upon relaxation, three of the four Cu atoms migrate towards the vacancy and rearrange to form a 3D triangle structure. The fourth atom remains at site **2**, which is located away from the vacancy, as shown in Figure 6G. The change in the binding energy is 0.39 eV compared to the Cu_4_ rhombus configuration at site **2** and 0.42 eV compared to the 3D triangle at the same site, making this structure the most favourable of the four-atom configurations. Cu–S distances are between 2.22 and 2.33 Å, while the Cu–Cu distances are between 2.33 Å and 2.35 Å. The Cu–S bonds are shorter compared to the 3D triangle at site **2** on the pristine surface, which has Cu–S distances between 2.31 and 2.46 Å, while the Cu–Cu distances are of similar length, ranging between 2.35 and 2.36 Å. The shorter Cu–S distances likely contribute to the more favourable binding energy. Similar to the stoichiometric monolayer, the stable tetrahedral configurations (at sites **2** and **3**, Figure 6H,I) are similar in energy, with a difference in stability of only 0.12 eV. Cu–S distances are between 2.29 and 2.36 Å and Cu–Cu distances are between 2.40 and 2.50 Å. These are some of the longest distances observed on the defective ML. However, they are somewhat shorter than those on the pristine surface, where the maximum Cu–S and Cu–Cu distances are 2.59 and 2.73 Å, respectively. This difference should contribute to the enhanced binding at the defective MoS_2_ ML.

The adsorption of four Cu atoms in a linear fashion (Figure 6E,F) is stable at both sites **1** and **2** on the defective ML, although these adsorption structures are not as favourable as the clustered structures, which is again due to the lack of Cu–Cu interactions. At site **1**, one of the four Cu atoms moves into the vacancy site, while two of four Cu atoms bind at the vacancy site for the site **2** configuration. These are the only two four-atom configurations where Cu–Mo interactions were observed, with distances between 2.62 and 2.65 Å. The Cu–S distances lie between 2.17 and 2.27 Å, while the Cu–Cu distances are 2.28 Å at site **1** and 2.29 Å at site **2**. Overall, the range of distances for Cu adsorption on the defective MoS_2_ monolayer is narrower compared to the range of distances on the pristine surface. Cu–Cu distances are of similar lengths to those measured for structures on the pristine surface, and are slightly shorter than the distances in bulk copper. As for the pristine surface, there is no clear correlation between the geometry and the strength of binding.

The presence of a defect did not cause any notable geometry distortions in the monolayer. This is supported by Mo–S distances at the copper adsorption sites, which lie between 2.38 and 2.49 Å, which is close to the Mo–S distance of 2.42 Å in bare MoS_2_. Some distortion was observed for the 3D Cu clusters (see Figure 6F,G in particular), which is caused by the migration of one or more Cu atoms towards the vacancy site.

The computed Bader charges determined for the Cu atoms on the defective monolayer are shown in Table 8. The computed Bader charge at the Cu atom at site **1** indicates little oxidation of the Cu atom. This arises due to coordination of Cu to three Mo atoms so that oxidation of the Cu is not favourable. The other binding sites have computed Bader charges consistent with the oxidation of Cu^0^ to Cu^+^, which are similar to the Bader charges computed for Cu adsorption at the bare monolayer, as detailed in Table 4 and Table 6.

**Table 8:** Computed Bader charge (*Q*) at each Cu atom for all Cu_1_ and Cu_4_ adsorption configurations on the MoS_2_ monolayer with an S vacancy. For the tetrahedral configuration, Cu_4 is at the apex, while the other three atoms are interacting with the monolayer. The rhombus structure rearranged and Cu_1 does not interact with the monolayer here.

site	configuration	*Q*(Cu_1)	*Q*(Cu_2)	*Q*(Cu_3)	*Q*(Cu_4)

1	single atom	10.87	—	—	—
2	single atom	10.52	—	—	—
3	single atom	10.49	—	—	—
					
1	line	10.89	10.85	10.88	10.84
2	line	10.81	10.83	10.66	10.63
2	rhombus	11.08	10.76	10.84	10.48
2	tetrahedral	10.71	10.65	10.66	11.05
3	tetrahedral	10.64	10.65	10.66	10.99

Comparing the different Cu_4_ adsorption structures, we find that in the two linear configurations the adsorbed Cu atoms are partially oxidised, with computed Bader charges of 10.84 to 10.89 electrons for the adsorption at site **1** and 10.63 to 10.83 electrons for the adsorption at site **2**. In the case of adsorption at site **2**, the two atoms closest to the vacancy (Cu_1 and Cu_2 in Table 8) are less oxidised than those further away. In the tetrahedral/triangular structures, the computed Bader charges for the Cu atoms directly bound to the monolayer are consistent with an oxidation to Cu^+^, while the remaining Cu atoms that do not interact with the monolayer are metallic. We further note that atoms are only partially oxidised when they are bound at the vacancy, while oxidation to Cu^+^ occurs for atoms further away from the vacancy. The same observations were also found for the pristine surface, indicating that the presence of the vacancy does not directly affect the charge transfer unless the Cu atom is adsorbed in or beside the vacancy site. Analysing the charge density difference for the two most favourable adsorption modes, shows that in contrast to the pristine surface the charge density is not limited to just adsorbed Cu atoms and the S and Mo atoms interacting directly with the Cu atoms. Instead, some charge density is delocalized to the S and Mo atoms neighbouring the S vacancy. Figure S5I and Figure S5H (Supporting Information File 1) show the charge density difference of the most favourable Cu*_n_* adsorption configurations on defective MoS_2_.

**Figure 7:**
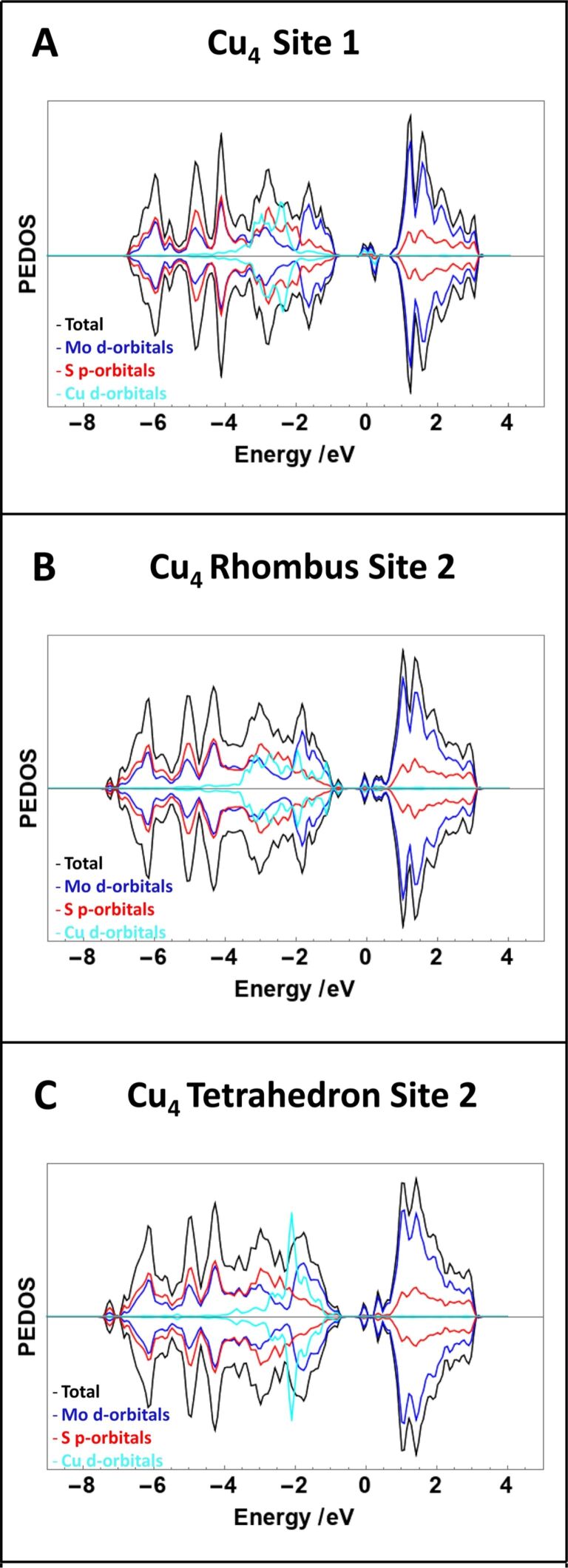
DOS plots of most favourable Cu_1_ and Cu_4_ adsorption structures on defective MoS_2_. The contribution of the Cu d orbitals has been increased by a factor of five for the ease of comparison and zero on the “energy” scale is the Fermi level.

Figure 7 shows the density of states for the three most favourable Cu adsorption configurations on defective MoS_2_. Density of states plots of all other adsorption configurations can be found in Supporting Information File 1, Figure S4. The general features of the DOS are similar to those of the pristine surface, in which mid-gap states originating from the presence of adsorbed copper can be seen for all configurations.

## Conclusion

The adsorption of metal species on semiconducting supports such as 2D monolayers of MoS_2_ is a subject of significant interest in a range of applications, particularly in catalysis and, more recently, in semiconductor nanodevices where 2D materials can function as barrier materials to prevent copper diffusion into the underlying dielectric material. While there have been studies of single-atom adsorption at MoS_2_ [26,29] and the adsorption of larger nanoclusters of noble metals, [25] there is as yet no comprehensive study of the interactions of small sub-nanometer metal species with a MoS_2_ ML, which is useful to probe the fundamental metal–MoS_2_ interactions. In this study, we investigated the adsorption behaviour of small Cu*_n_* nanoclusters (*n* = 1–4) through first-principles density functional theory.

We find that a single Cu atom prefers to adsorb above a Mo atom, compared to adsorption atop S or in the hollow site of the Mo–S hexagon. However, as *n* increases, the effect of the adsorption site on the binding energy becomes less important, although the atoms remain at their original adsorption site throughout the relaxation. Interestingly, Cu_4_ clusters seem to prefer to adsorb with the Cu atoms atop the S atoms, even though this is the least favourable adsorption site for a single atom. This can be attributed to the shorter distance between Cu and S, which facilitates the formation of more Cu–Cu bonds.

Bader charge analysis shows that Cu atoms interacting with the MoS_2_ ML are oxidised to Cu^+^, while the apex atoms in the 3D structures, which only interact with other Cu atoms, remain as metallic Cu^0^ species. Overall, the relative stability of a Cu*_n_* adsorption structure is driven by the Cu–Cu interactions, which are in turn promoted by the distance between adsorption sites. This leads to 3D Cu structures having similar binding energies regardless of adsorption site. Further, there is no real preference between the 2D Cu_4_ rhombus structure, which has the largest number of Cu–Cu bonds of any of the 2D structures, and the two 3D Cu_4_ structures studied.

Removing a single S atom from the MoS_2_ monolayer, which is highly favourable, enhances the binding of Cu nanoclusters to the MoS_2_ ML. The effect of the vacancy is found to be localised and charge transfer follows the same trend as on the pristine surface unless a Cu atom adsorbs in the vacancy site, which is a highly favourable process; in this case oxidation of the Cu atom is less pronounced.

Although there are many Cu_4_ adsorption structures that are stable on the pristine ML, they are no longer stable upon adsorption at the defective ML. There is a preference for 3D structures on the defective MoS_2_ ML. In future work, larger Cu*_n_* structures will be required to explore if Cu grows as a 2D Cu film or prefers to form 3D clusters and how this can be tuned by the stoichiometry of the MoS_2_ ML.

## Supporting Information

File 1 Additional computational data
