# Supplementary material for: DFT calculations of the structure and stability of copper clusters on MoS2
Source: Beilstein J Nanotechnol. 2020 Feb 26;11:391–406. doi: 10.3762/bjnano.11.30 (PMC7059439; doi:10.3762/bjnano.11.30)
Supplement: File 1 — Additional computational data [file Beilstein_J_Nanotechnol-11-391-s001.pdf]

BEILSTEIN JOURNAL OF NANOTECHNOLOGY

Supporting Information

for

DFT calculations of the structure and stability of copper clusters on MoS_2

Cara-Lena Nies and Michael Nolan

Beilstein J. Nanotechnol. **2020,** *11,* 391–406. doi:10.3762/bjnano.11.30

Additional computational data

License and Terms: This is a supporting information file under the terms of the Creative Commons Attribution License (https://creativecommons.org/licenses/by/4.0). Please note that the reuse, redistribution and reproduction in particular requires that the authors and source are credited.
The license is subject to the *Beilstein Journal of Nanotechnology* terms and conditions: (https://www.beilstein-journals.org/bjnano)

DOS Plots

Density of state plots for all Cu_n adsorption configurations are given below.

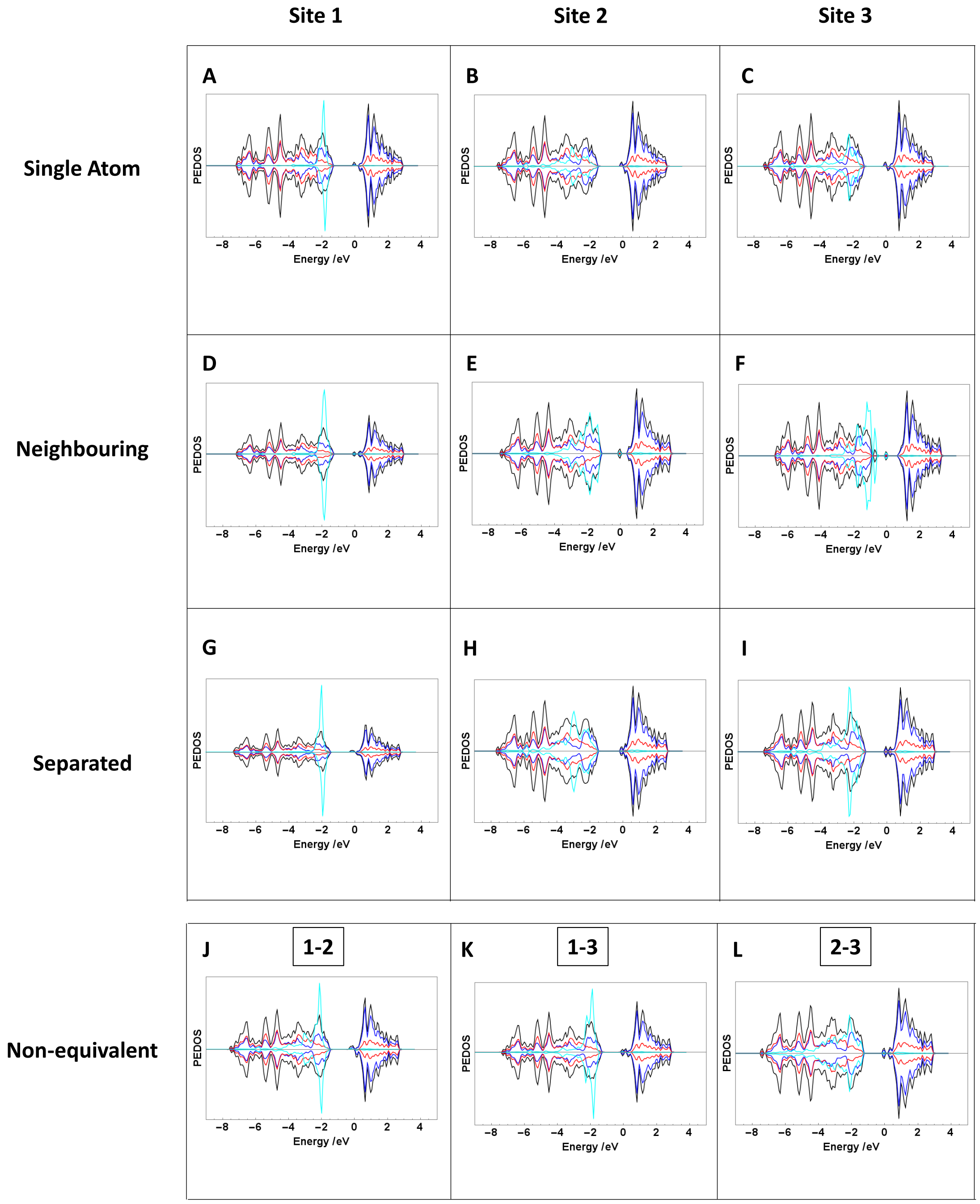

Figure S1: DOS plots of Cu_1 and Cu_2 adsorption structures. Black = total DOS, blue = DOS projected onto Mo d orbitals, red = DOS projected onto S p orbitals, cyan = DOS projected onto Cu d orbitals. The contribution of the Cu d orbitals has been increased by a factor of 5 for the ease of comparison.

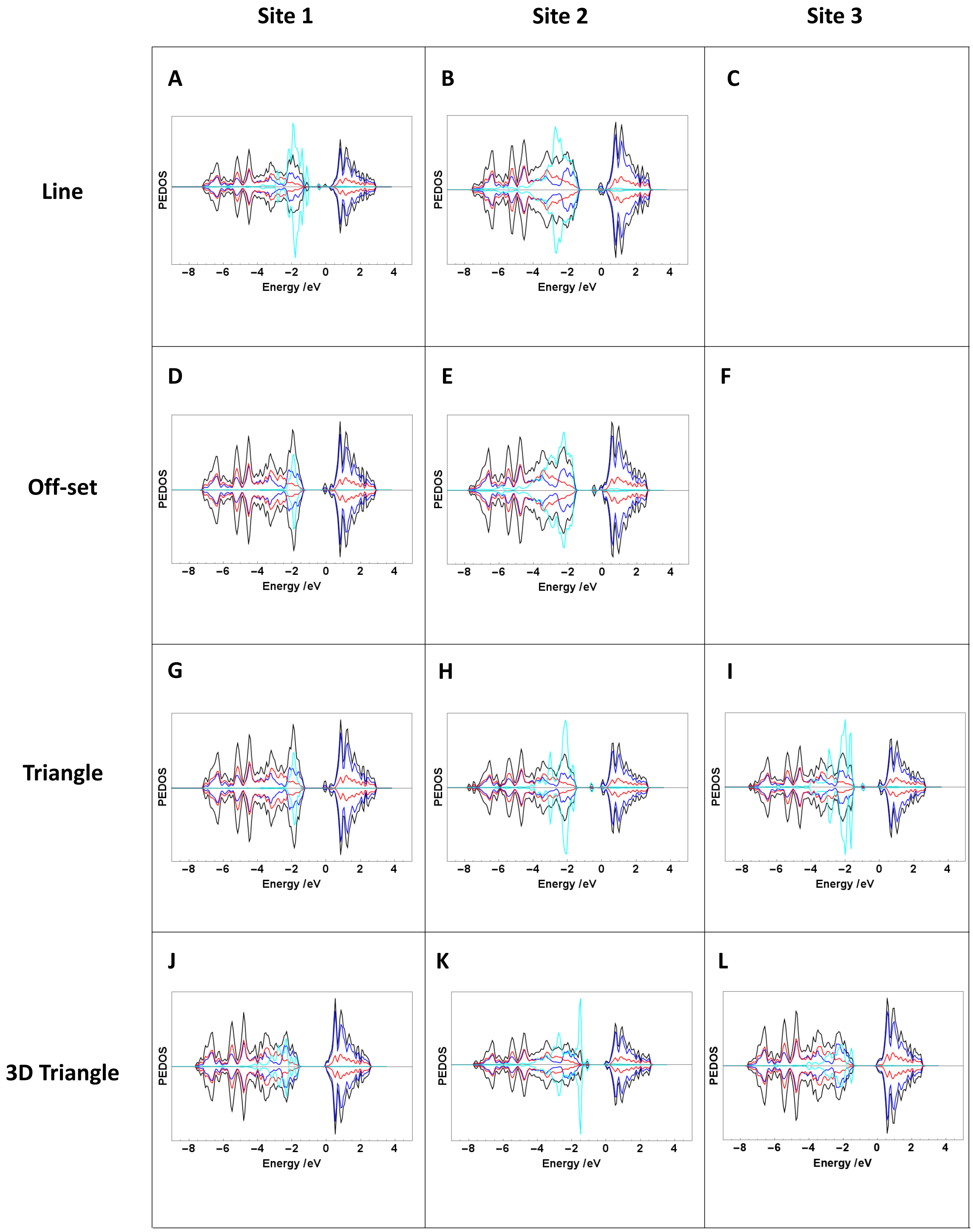

Figure S2: DOS plots of Cu_3 adsorption structures. Black = total DOS, blue = DOS projected onto Mo d orbitals, red = DOS projected onto S p orbitals, cyan = DOS projected onto Cu d orbitals. The contribution of the Cu d orbitals has been increased by a factor of five for the ease of comparison.

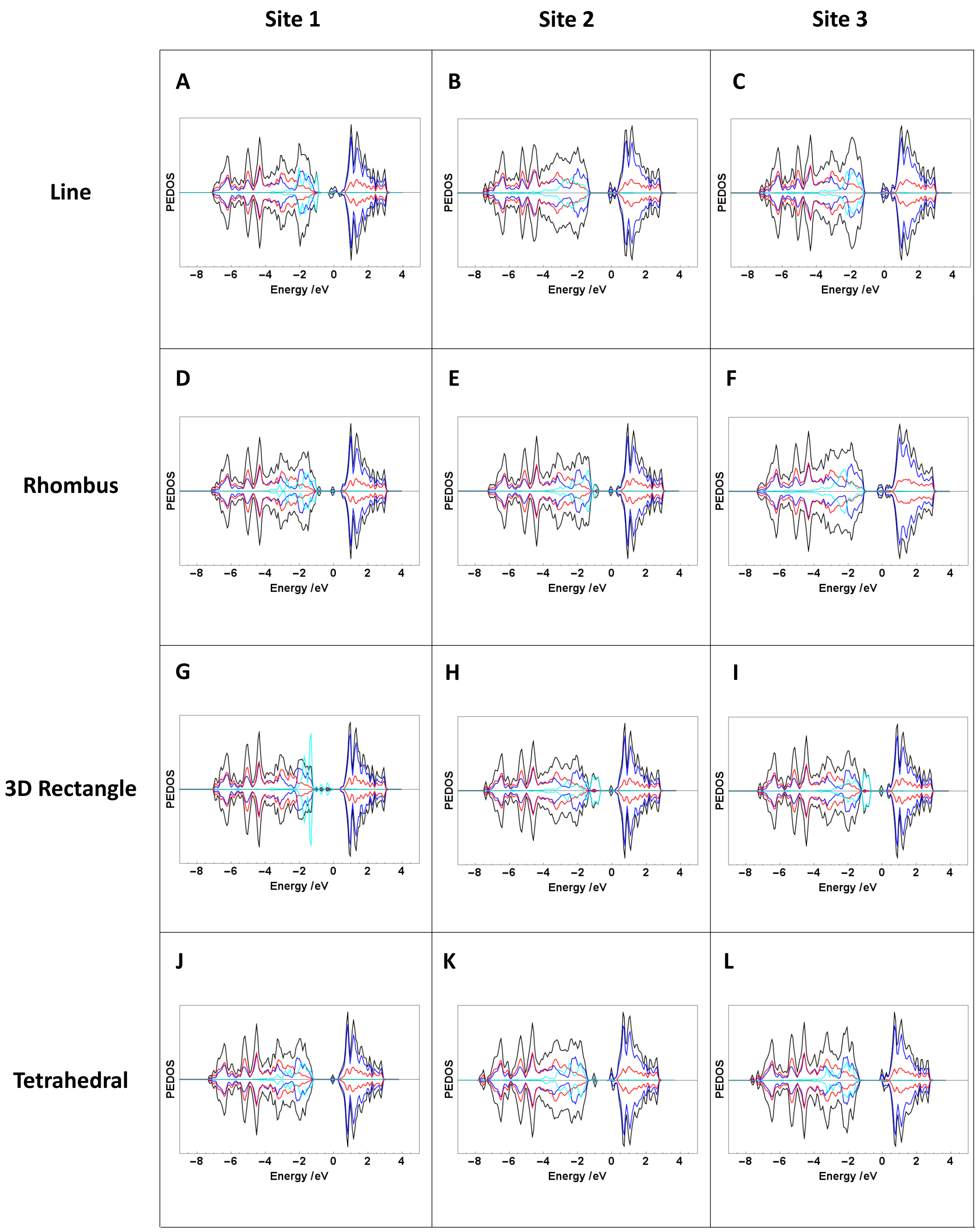

Figure S3: DOS plots of Cu_4 adsorption structures. Black = total DOS, blue = DOS projected onto Mo d orbitals, red = DOS projected onto S p orbitals, cyan = DOS projected onto Cu d orbitals. The contribution of the Cu d orbitals has been increased by a factor of five for the ease of comparison.

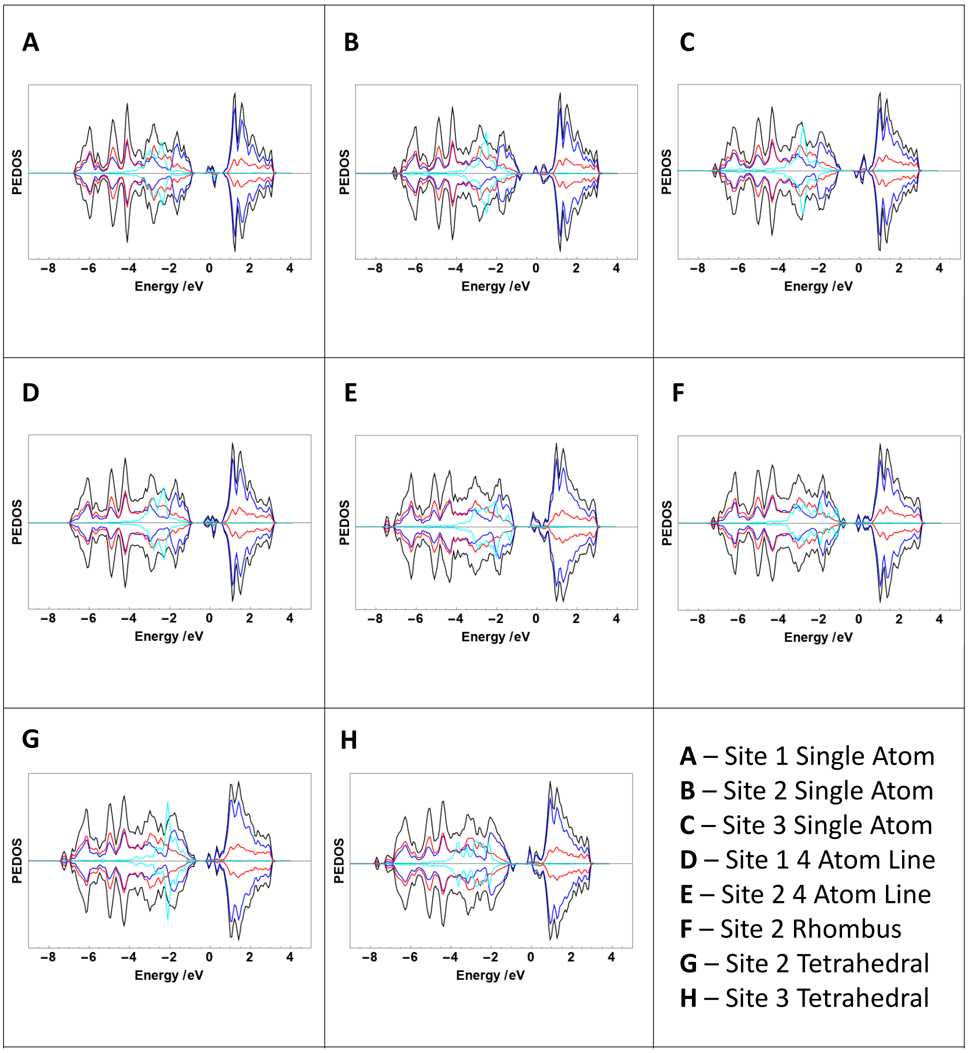

Figure S4: DOS plots of Cu_1 and Cu_4 adsorption structures on defective MoS_2. Black = total DOS, blue = DOS projected onto Mo d orbitals, red = DOS projected onto S p orbitals, cyan = DOS projected onto Cu d orbitals. The contribution of the Cu d orbitals has been increased by a factor of five for the ease of comparison.

Charge Density Distribution after adsorption of Cu_n

The charge density difference after adsorption of the most favourable Cu_n is shown below.

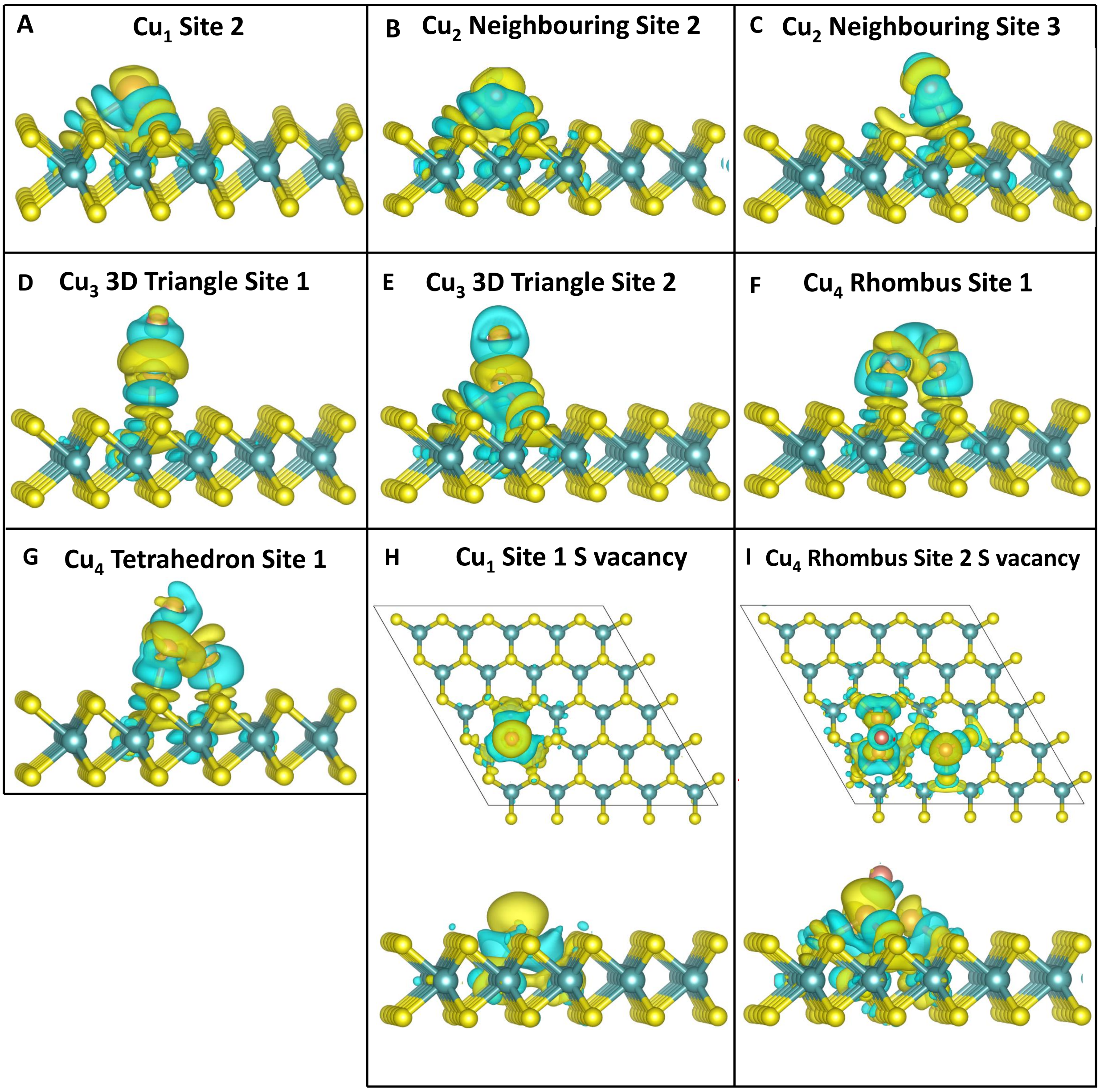

Figure S5: Charge density difference after adsorption of the most favourable Cu_n on pristine (A–G) and defective (H, I) MoS_2.

Cu atom repelled from ML

An example of a Cu atom that is repelled from the MoS_2 ML is shown in Figure S6. This occurred during the relaxation of several Cu_4 structures on the MoS_2 monolayer with an S vacancy.

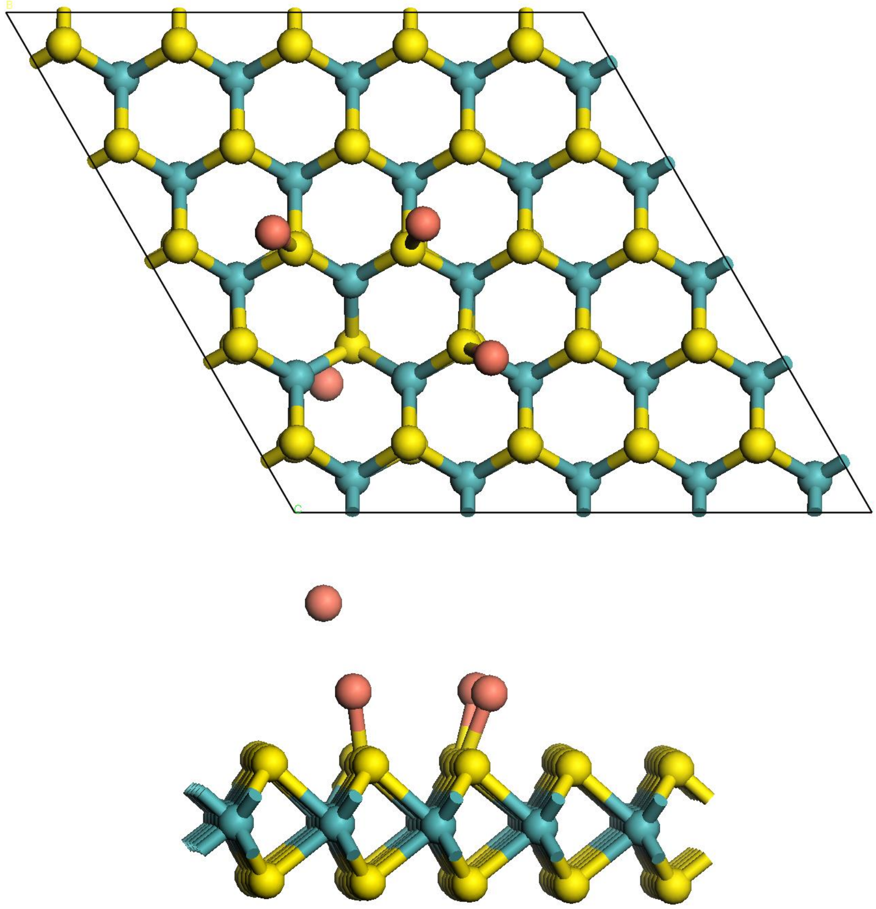

Figure S6: A Cu atom is repelled from the surface during the geometry relaxation.
